# The 2-week wait pathway for suspected head and neck cancers in patients with throat and voice symptoms: referral patterns, common clinical practice and diagnostic efficacy of NICE guidelines

**DOI:** 10.1308/rcsann.2024.0072

**Published:** 2024-11-12

**Authors:** FA Chiumenti, AL Pendolino, FM Vaz, RC Dwivedi

**Affiliations:** ^1^ University College London Hospitals NHS Foundation Trust, UK; ^2^ UCL Ear Institute, UK; ^3^ Christchurch Canterbury University, UK; ^4^ University College London, UK

**Keywords:** Head and neck cancers, 2-week wait pathway, NICE guidelines, Throat and voice symptoms, Risk stratification, Laryngopharyngeal reflux, Proton pump inhibitors, Psychological distress

## Abstract

**Introduction:**

The 2-week wait (2ww) referral pathway has been introduced into UK clinical practice to increase the early detection of cancer and improve survivals. The efficiency of this system for head and neck (H&N) cancers has been questioned over the years because of evidence of low pick-up rates. H&N cancers present with a wide variety of non-specific symptoms, particularly throat and voice symptoms. These symptoms need to be accurately interpreted together with risk factors if they are to be addressed adequately and overload of cancer facilities avoided. One of the most common outcomes of H&N 2ww referrals is laryngopharyngeal reflux (LPR), a common condition that could be diagnosed and managed in the primary care setting with a prescription of proton pump inhibitors (PPI) trials.

**Methods:**

We retrospectively analysed a cohort of consecutive patients referred on the 2ww pathway for throat and voice symptoms at University College London Hospital H&N cancer clinic during two months in 2019.

**Results:**

A total of 101 patients (43.6% men, mean age 53.3 years) were included. Throat and voice symptoms were described as intermittent in 52.5% and non-lateralised in 88.1%. Diagnosis of LPR was made in 59.4% of the referrals. A PPI trial was prescribed by general practitioners (GPs) in only 7.9% of cases. The cancer pick-up rate in our cohort is 2.9%.

**Conclusions:**

An improved awareness of the symptoms of LPR could guide GPs to prescribe trials of PPIs in low-risk patients before rushing into a referral on a cancer pathway. This would improve the 2ww process on many levels, reduce the burden on the National Health Service and avoid patients' psychological distress.

## Introduction

Head and neck (H&N) cancers represent 3% of cancers in the UK, with 12,422 new patients diagnosed every year.^1^ Laryngeal cancer is the second most common H&N cancer, and is responsible for >159,000 new cases and approximately 90,000 cancer deaths each year worldwide.^2^ Hypopharyngeal cancers, although rare, have persistently low survival rates (1, 5 and 10 years survival 60.5%, 27.8% and 19.1%, respectively), potentially because of overlooked symptoms leading to delayed diagnosis at an advanced stage.^1^

The importance of early referral of patients with symptoms suspicious for cancer was first addressed in 1999, when the Department of Health introduced the 2-week wait (2ww) referral pathway.^3^ The aim of the protocols was to identify patients at risk and promptly refer them to specialist care. The latest version of the guidelines for H&N cancer referrals was issued in June 2015 by the National Institute for Health and Care Excellence (NICE).^4^

Several audits across the country have, however, repeatedly highlighted the low detection rates of the H&N 2ww referral system.^5–10^ This has caused an overload in National Health Service (NHS) secondary and tertiary care facilities, especially in the post-Covid period when there has been a rise in 2ww H&N referrals.^11^

Symptoms that are regarded as suspicious of throat cancer can be non-specific and overlap with those of many inflammatory conditions. To produce an effective referral, symptoms need to be related to their duration and localisation, and to each patient's respective risk factors.

One common chronic inflammatory disease of the upper aerodigestive tract is laryngopharyngeal reflux (LPR). The prevalence of LPR has been explored in different populations and it is reported to be between 10% and 18%.^12–14^ In the English population LPR prevalence is as high as 34.4%.^15^ LPR symptoms include the sensation of something stuck in the throat, hoarseness, frequent throat clearing, throat dryness and soreness, excessive phlegm and chronic dry cough. The Reflux Score Index (RSI) is a validated tool to assess LPR symptoms.^16^ It can be used both for diagnosis and follow-up. A descriptive cross-sectional study conducted in two tertiary care hospitals showed RSI to have sensitivity and specificity of 79.1% and 83.7%, respectively, with an accuracy of 81.8%.^17^

The Reflux Sign Assessment (RSA) score describes LPR-induced signs both detected endoscopically and on examination of oral cavity and oropharynx (eg anterior pillar and uvula erythema, coated tongue and oropharyngeal posterior wall inflammatory granulations).^18^ The RSA has shown high intra- and interrater reliabilities and responsiveness to change.^18^ Using both tools can improve accuracy and reliability at the time of first assessment and of re-evaluation after treatment.^18^ Moreover, the combination of a short version of the RSA (RSA-10) and RSS-12 (Reflux Symptom Score) has been shown to have sensitivity, specificity, positive and negative predictive values of 92.7%, 97.3%, 94.4%, and 96.5%, respectively.^19^

Both scores were validated in tertiary care facilities in which, particularly for RSA, laryngoscopy and its interpretation from a specialist are available. However, what RSA adds to previous signs scores (eg the Reflux Finding Score) is the evaluation of oral and oropharyngeal changes that can be identified in a primary care setting without the need for endoscopic investigation. The use of RSA in the context of a detailed algorithm to be adopted in the primary care setting has been already proposed by the creator of the tool.^20^ Adopting these tools in general practice could allow LPR management to be started in the primary care setting.

First-line treatment of LPR consists of dietary and lifestyle adjustments, eventually complemented by medications such as non-prescription sodium alginate preparations and/or a 4-week course of proton pump inhibitors (PPIs). An empirical trial of PPIs is a reasonable first-line management in low-risk patients presenting with throat and voice symptoms suggestive of LPR, significantly improving them.^21,22^

Systematic reviews and meta-analysis have reported mixed results regarding the efficacy of PPIs in LPR treatment. This has shown to be related to interstudy variability of dosage, frequency, diagnostic and inclusion criteria, and the pathophysiology of the underlying disease.^23^

The TOPPITS randomised controlled trial studied the effects of PPIs on patients with persistent throat symptoms and found no significant difference in improvement of RSI between the treatment and placebo groups. Interestingly, this study did not include patients with a formal diagnosis of reflux-related disease (pH monitoring) and excluded patients with ‘heartburn’.^24^ These exclusion criteria might have led to the omission of patients with acidic reflux. It has been hypothesised that acidic reflux might be more commonly associated with classic reflux symptoms (heartburn, belching) instead of isolated throat symptoms.^25^ Therefore, the absence of a significant effect of PPIs might be related to some of the included patients suffering from non-acidic or mixed reflux.

Sodium alginate preparations create a mechanical barrier to reflux, therefore acting both on acidic and non-acidic events. They can be prescribed as an adjunct to PPIs to increase the chance of symptom improvement in empiric treatment, either as a first- or second-line measure.^23^

## Methods

We present a retrospective analysis on a cohort of consecutive patients that were referred on a 2ww pathway to University College London Hospital (UCLH) ear, nose and throat (ENT) H&N department outpatient clinic between 2 October and 30 November 2019. Referrals were made by general practitioners (GPs) based on the Pan London Suspected Head and Neck Cancer Referral Form (Figure 1). Patients’ data were reported and collected on UCLH Epic software.

**Figure 1:**
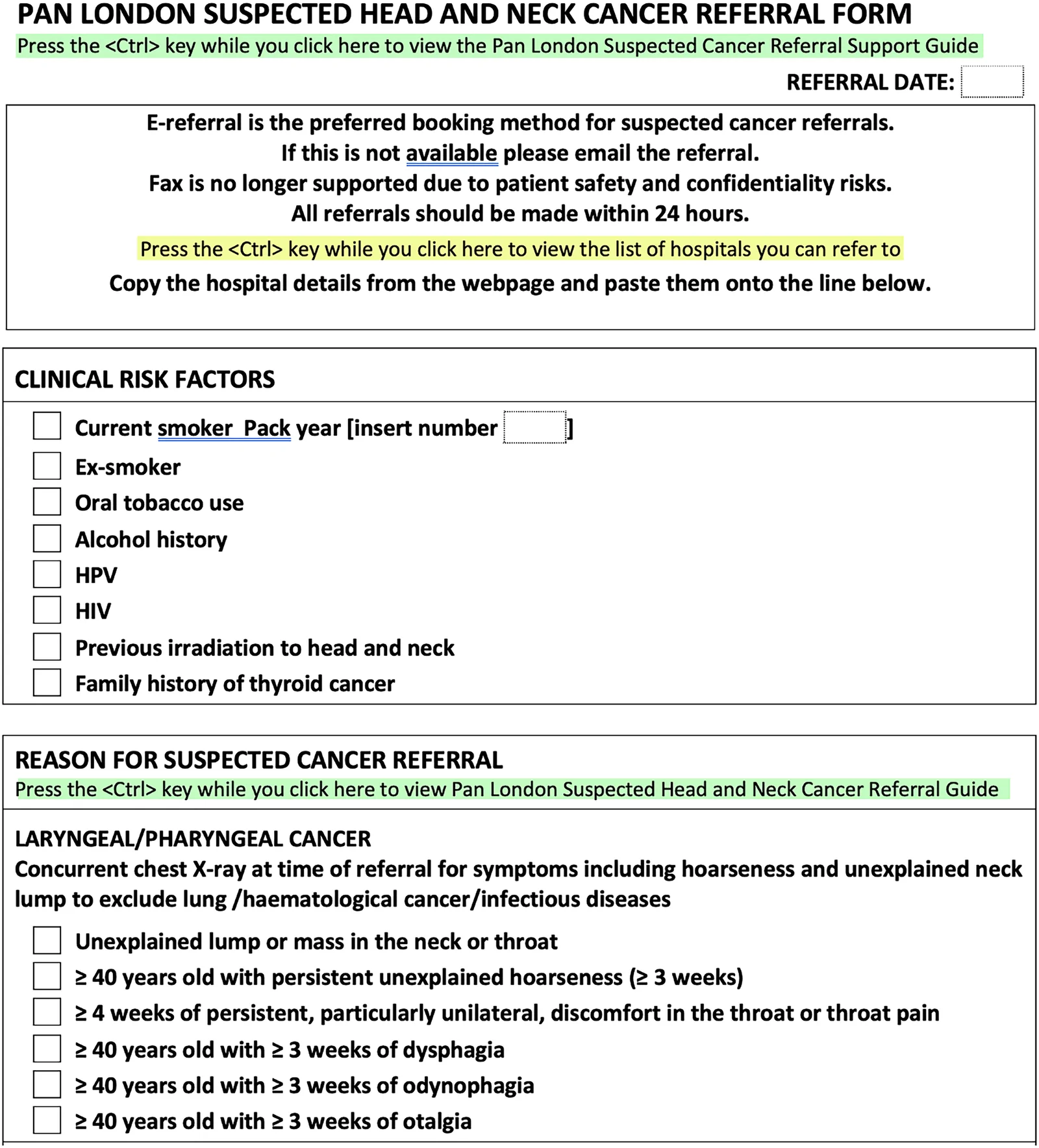
Extract of clinical risk factors and pharyngeal/laryngeal symptoms from Pan London Suspected Head and Neck Cancer Referral

We included patients referred with throat symptoms, namely throat discomfort, soreness and dryness, hoarseness, feeling of something stuck in throat, dysphagia, odynophagia and chronic cough. Exclusion criteria were patients referred with neck/thyroid lumps, maxillofacial and oral lesions and ear/nose symptoms.

Symptoms were described as generalised or unilateral and intermittent or persistent (the latter when they were ongoing for three or more weeks, as per NICE guidelines). We regarded as high-risk patients those who were current or ex-smokers, with an active or former alcohol consumption of more than 14U/week, betel nut products chewers, patients with personal or family history of cancer or dysplastic lesions in the H&N region, and patients with low immunity.^1,26^

### Ethics approval

This study was conducted as a part of an audit to improve service at our department and it has been registered at the institutional audit committee. Ethics approval was not required.

## Results

A total of 214 patient referrals were made to the ENT H&N clinic at UCLH under the 2ww pathway during the analysed two-month period. We included 101 consecutive patients that fulfilled the inclusion criteria.

The general characteristics of the study population, symptoms and clinic outcomes are reported in Table 1.

**Table 1:** General characteristic of the population

Characteristic	*N* = 101
Age, mean [range], years	56.3 [27–95]
≥40	83 (82.2)
<40	18 (17.8)
Sex	
Female	57 (56.4)
Male	44 (43.6)
High-risk patients	48 (47.5)
Duration of symptoms	
Persistent	46 (45.5)
Intermittent	53 (52.5)
None (symptoms resolved)	2 (2.0)
Laterality	
Bilateral	89 (88.1)
Unilateral	12 (11.9)
Treatment with PPI	
On long-term PPI	22 (21.8)
Started by GP	8 (7.9)
Prescribed/adjusted in clinic	59 (58.4)
Not needed	12 (11.9)
Need for further investigations	33 (32.7)
Surgical indication	7 (6.9)
Diagnosis	
Benign/functional pathology	23 (22.8)
LPR	60 (59.4)
Malignancy^a^	3 (2.9)
No H&N pathology	15 (14.9)
Re-referred once discharged	2 (1.9)

GP = general practitioner; H&N = head and neck; LPR = laryngopharyngeal reflux; PPI = proton pump inhibitor

Values are given as n (%) unless stated otherwise

^a^One patient had an incidental diagnosis of papillary thyroid cancer and it was not included in our analysis.

Two patients (1.9%) were incidentally diagnosed with thyroid nodules and underwent thyroid surgery. One was diagnosed with a papillary carcinoma. We did not include this in our analysis.

Data on benign and functional pathologies diagnosed in the 2ww clinic are reported in Table 2.

**Table 2:** Presenting symptoms, investigations needed and type of treatment for the patients with benign and malignant head and neck pathologies diagnosed in 2-week wait head and neck clinic

	No. of patients (%)	Presenting symptom(s)	Further investigation(s)	Treatment
Benign pathology	*n* = 23			
Vocal cord palsy^a^	6 (26.1)	Hoarseness	MRI neck + CT chest	2 augmentations 4 conservative (good compensation)
Vocal cord lesion^b^	6 (26.1)	Hoarseness	—	Microlaryngoscopy and biopsy
Tonsil lesion^c^	1 (4.3)	FOSSIT	—	Biopsy
Functional UADT pathology^d^	5 (21.7)	FOSSIT, sore throat, intermittent dysphagia	Barium swallow	—
Neurological causes^e^	2 (8.7)	Hoarseness, FOSSIT, choking	Barium swallow	Speech and language referral
Inflammatory causes^f^	3 (13.0)	Hoarseness, dry cough, sore throat	—	Conservative
Malignancy (TNM)	*n* = 3			
Tongue base (T2N2bM0 scc p16-)	1 (25.0)	Unilateral sore throat and odynophagia	US neck + FNAC, MRI neck, CT chest	Radical RT 65G/30#
Tonsil (T4aN0M0 scc p16-)	1 (25.0)	Right otalgia and odynophagia	US neck + FNAC, MRI neck, CT chest	Radical RT 65G/30#
Supraglottis (T3N2bMx scc p16-)	1 (25.0)	Hoarseness	US neck + FNAC, MRI neck, CT chest	Palliative RT 27G/6# (lung cancer)

CT = computed tomography; FNAC = fine needle aspiration cytology; FOSSIT = feeling of something stuck in throat; MRI = Magnetic resonance imaging; UADT = upper aerodigestive tract; US = ultrasound

^a^Vocal fold palsies were idiopathic in five cases, one was probably related to a foramen magnum decompression for Chiari malformation.

^b^Vocal cord lesions included polyp, dysplasia, Reinke’s oedema and squamous papilloma

^c^Tonsil lesion was reported as a fibrous polyp on histopathological examination

^d^Functional UADT pathologies included: cricopharyngeal spasm, oesophageal dysmotility and hiatus hernia

^e^Neurological causes include: familial tremor and fluid/food aspiration

^f^Inflammatory causes include: laryngitis and vocal process granuloma

Diagnosis of LPR was made in 59.4% of patients. Twenty-two patients (21.8%) were already on longstanding PPI medication. Eight patients (7.9%) were given a trial by their GP before the 2ww referral. In 16 patients (14.8%), the H&N specialist had either increased the dosage of the same PPI, changed the PPI molecule (to overcome drug tolerance in cases of longstanding treatment) or added a sodium alginate reflux suppressant. In 43 patients, 71.7% of those diagnosed with LPR, PPI drug and or reflux suppressant was prescribed for the first time after clinic appointment.

In the entire cohort only three patients were diagnosed with laryngeal or pharyngeal cancer, which translates to a pick-up rate of 2.9%. They were all high-risk patients (smokers, presenting with unilateral, persistent and worsening symptoms of odynophagia and ipsilateral otalgia, developed over 1 or 2 months before referral). These patients were all diagnosed with advanced stage diseases and underwent radiotherapy (RT) or chemoradiotherapy (CRT) with curative or palliative intent. They all died from local recurrence or disease progression (Table 2).

## Discussion

### Summary

H&N cancers are the eighth most common malignancies in the UK. Incidence has been on the rise since 2009, being highest in men in their seventh decade. The mortality rate has overall seen a decrease in its figures.^1^ One of the most important prognostic factors in H&N cancer is the stage of the disease at diagnosis. This renders early detection a primary goal of H&N cancer management. The aim of the Department of Health legislation that in 1999 introduced the 2ww referral pathway in the UK was to increase the efficacy of NHS in early cancer diagnosis and treatment, to subsequently improve survival rates.

All over the country H&N clinics have implemented their workload and showed a very good compliance to meet fast-track referral criteria. Efficiency rates in meeting the 2-week timespan to review suspected cancer patients are reported to be between 95% and 100% in different studies.^6–8,10^ Proof of the positive role of H&N clinics, that to the best of our knowledge had not been previously reported in the literature, is the very low rate of re-referrals that we observed in our cohort after patient discharge (1.9%); only two patients were re-referred and discharged with the same symptoms of LPR. This also suggests that no cancer diagnoses have been missed.

Nonetheless, data from national audits disclose a low threshold in primary care for the use of the 2ww route.^5–10^ This might be related to a degree of clinical uncertainty regarding H&N pathologies and poor awareness on LPR and tools to assess it, as described by different surveys.^27,28^ Improving communication between primary and specialist care clinicians and implementing educational programmes have both been suggested in the past.^5,10^ However, current NHS overload and financial crisis might not allow such measures. Future research with a direct collaboration between GPs and ENT to test the effectiveness of ameliorated risk stratification and prescription of empiric treatment should be encouraged.

Over-referral might also be caused by patient pressure. Often it is unclear whether patients actually meet the criteria for referral, or the fast-track is used to guarantee a quick clinic appointment or to avoid medico-legal issues.

Two more aspects can explain the low cancer pick-up rates associated with throat and voice symptoms. One is the vagueness of words included in NICE guidelines definitions (Figure 1).^4^ Williams *et al* expressed their concern over a cancer pick-up rate of 1.1% for patients referred for “unexplained, persistent sore throat”.^6^ Hong observed how despite 93.6% 2ww referrals meeting NICE criteria, relative malignancy pick-up rate was only 6.2%.^10^

The second component is the very large spectrum of pathologies, mainly non-concerning inflammatory ones, that can lead to throat symptoms. In a large cohort of more than 1,000 patients, McKie *et al*^7^ found that throat symptoms were the most common reason for urgent referral to an H&N specialist and, after clinic appointment, those were largely attributed to reflux and globus phenomenon. Our data are not dissimilar, with 59.4% of 2ww referrals being diagnosed with LPR.

LPR symptoms often come in clusters and once acknowledged can be readily addressed with dietary and lifestyle changes with or without prescription of PPIs and alginate solutions. PPIs are generally safe and manageable medications, mostly with mild side effects and, if appropriately prescribed, they can improve LPR symptoms.^21,22,27^ Eight weeks is the minimum recommended treatment to allow mucosal healing and regeneration;^29^ however, in the context of needing to rule out more sinister pathologies, we believe 4 weeks would be enough time to reassess the patient for any improvement.

In our series, 7.9% of patients were prescribed a PPI trial before the 2ww referral and at their clinic appointment LPR diagnosis and treatment were confirmed. The majority of patients (92.1%) did not have any trial of PPI and were referred directly using 2ww referral pathway.

One more aspect that should be taken into account when considering the inappropriate use of 2ww referral on a cancer pathway is the psychological burden on patients. NICE guidelines strongly recommend that GPs inform patients about the reasons for any urgency and involve them in the final decision of their referral on a cancer pathway. In such a context, patients awaiting H&N clinic appointments can experience considerable anxiety, distress, fear and vulnerability. Psychological impact during diagnostic phase for suspected H&N cancer and how the referral was conducted have been investigated in only two studies.^10,30^ One study reports anxiety prior to clinic appointment in 77.2% of patients and that only 27.7% were told about provisional or differential diagnoses.^10^ Rowlands *et al* found that 58% of patients reported a degree of concern related to the referral, and high levels of worry were more common in women and in patients under 40 years of age.^30^

### Comparison with other studies

Fast-track referrals are now part of current clinical practice and their efficacy has been widely examined. Previous studies have highlighted a continuous increase in referral rates over time and conversely a reduction of cancer pick-up rates.

Williams *et al* made a comparison of 2ww referrals features between two separate periods, 10 years apart, in the same hospital.^6^ Their data show how fast-track referrals increased fourfold whereas cancer diagnosis among those referrals fell by half. The literature has historically reported a pick-up rate of H&N cancers from 2ww referrals of between 10% and 15%;^6–8^ however, most recent studies show a concerning fall in this rate. In their 2012 cohort, Williams *et al* found a pick-up rate of 5%;^6^ in 2016 Hong *et al* found a 6.3% rate of cancer diagnosis from fast-track referral, and in 2019 the same rate was 3.7% in the study by Gao *et al*.^5,10^ In our study, the pick-up rate is even lower (2.9%). Direct comparison cannot, however, be made because we did not include all 2ww referrals in our cohort and our study period is relatively shorter.

We focused on throat and voice symptoms, the most common reason for referral for suspected H&N cancers. In a large cohort of 984 patients Gao *et al* report that 47.3% of all 2ww referrals were for throat symptoms, namely “persistent unexplained hoarseness” and “persistent, particularly unilateral, throat discomfort”.^5^ None of these symptoms yielded a significant odds ratio in the study results. Tikka *et al* also highlighted how hoarseness and a sensation of lump in the throat are negatively related with cancer diagnosis, and therefore, mostly when they present as a sole symptom, discourage a 2ww referral.^31^

Risk stratification of patients is essential to appropriately address symptoms. A correlation between red flags, patient risk factors and demographics to generate a scoring system has previously been suggested.^9,31^ In our series, only 6.9% of cases were both considered high-risk patients and had concerning features related to their symptoms (unilateral odynophagia with or without otalgia). Among these were all three cancer diagnoses of the series. Gao *et al*^5^ stratified patient risk factors and findings for those patients deemed to be of low clinical risk; all proved to be negative for cancer.

### Strengths and limitations

This is a pilot study on this topic and therefore the sample size is relatively small. However, our results clearly show that H&N 2ww referral pathways are being inefficiently utilised and there is scope for improvement in this.

A strong point that emerges from our series is that not only is the pick-up rate very low, but also all patients diagnosed via the 2ww pathway had advanced disease with non-favourable outcomes and had lateralised symptoms or were high risk to begin with. This should induce reflection upon the need for improved patient and primary care awareness.

This appears to be the first dedicated study to analyse throat and voice symptoms separately from other H&N complaints, looking specifically into suspected laryngeal and pharyngeal cancers among 2ww H&N referrals. With this work we are trying to raise awareness of LPR and its possible management in the primary care setting when high-risk criteria are not met. We also hope that our work will generate a thought process among other colleagues so that larger multicentre studies can be set up with application of risk stratification in the primary care and trial treatments.

### Implication for practice

Targeted education programmes for clinicians and modification of the Pan-London H&N cancer 2ww referral to increase its efficacy have previously been suggested^5^ and we are in strong agreement.

We propose a straightforward algorithm for the management of patients presenting with throat and voice symptoms, hoping this will help improve the efficiency of the 2ww pathway, reduce the burden on specialised centres and avoid patients' psychological distress (Figure 2). For low-risk patients, we suggest GP prescription of a 4-week bridging trial of PPIs with or without sodium alginate solutions, along with lifestyle and dietary modifications before specialist referral in selected patients. This is based on awareness around concerning features of throat and voice symptoms (ie unilaterality and persistence with worsening trend over a relatively short period) and familiarity with H&N cancer risk factors (smoking, alcohol, premalignant conditions, previous irradiation, acquired or inherited immunodeficiency), to allow adequate risk stratification.

**Figure 2:**
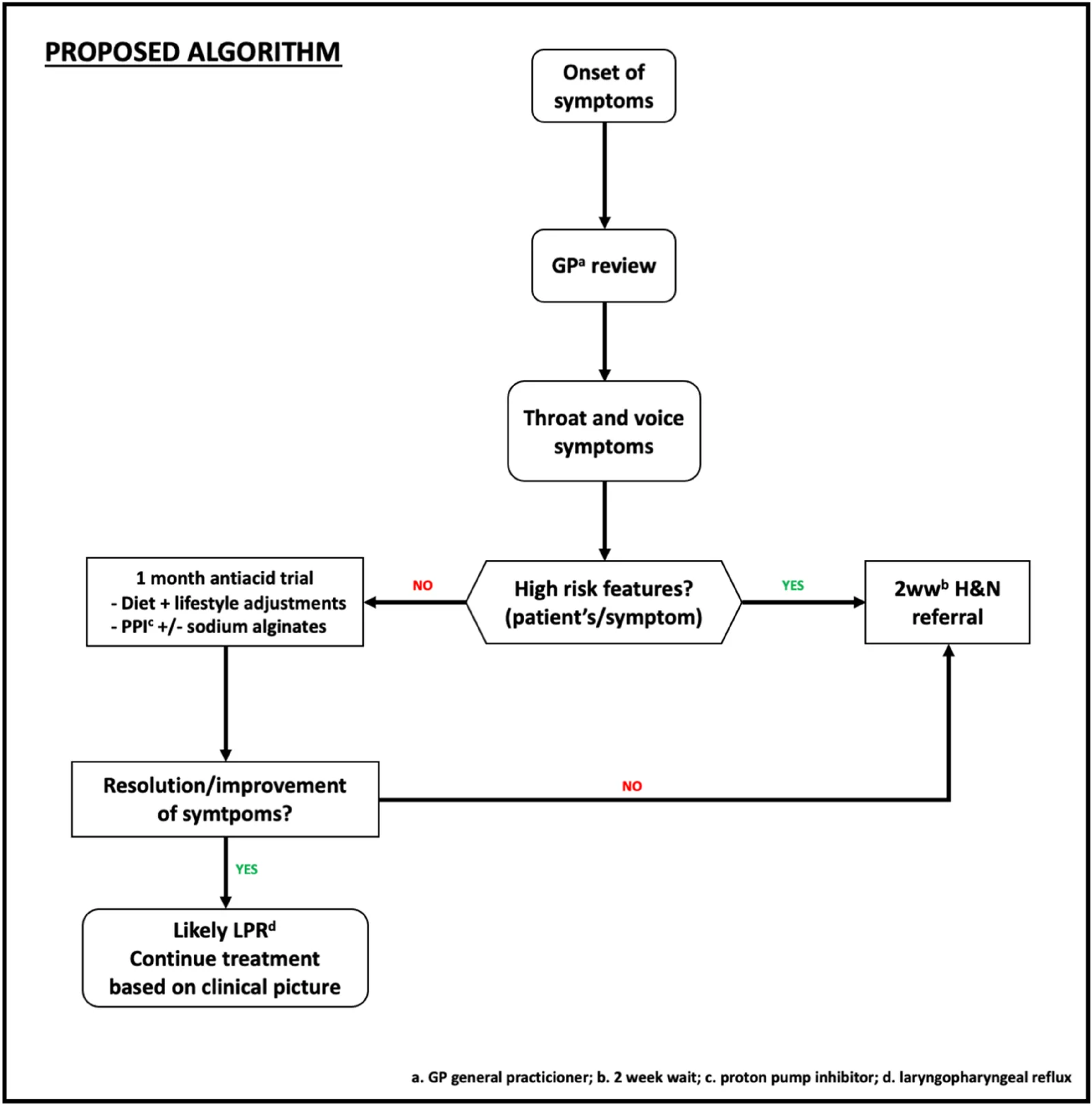
Proposed algorithm for primary care management of throat and voice symptoms based on risk stratification and antiacid treatment trial

## Conclusions

Our experience shows a low pick-up rate of 2ww referrals for laryngeal and pharyngeal cancer. A high proportion of 2ww referrals are made for LPR. LPR is easily detectable based on symptoms and examination of the oral cavity and oropharynx and can be conducted in the primary care setting. An improved awareness of LPR among GPs and an agreed modification in clinical practice in the form of a therapeutic trial in low-risk patients could sensibly improve the whole 2ww process. It would reduce the rate of inappropriate referrals, improve pick-up rates, lighten the pressure on secondary and tertiary centres, and enhance allocation of resources and patient care across the UK, especially when the healthcare system is trying to deal with post-Covid backlogs and an ongoing financial crisis.

Current NICE guidelines need careful consideration and amendment to be more direct, specific and clear about high-risk symptoms and patients for more effective use of fast-track pathways.

Moreover, modification of the primary care approach would avoid unnecessary psychological distress to patients who are dealing with the current period of austerity and uncertainty.
